# Brain-Specific Superoxide Dismutase 2 Deficiency Causes Perinatal Death with Spongiform Encephalopathy in Mice

**DOI:** 10.1155/2015/238914

**Published:** 2015-08-02

**Authors:** Naotaka Izuo, Hidetoshi Nojiri, Satoshi Uchiyama, Yoshihiro Noda, Satoru Kawakami, Shuji Kojima, Toru Sasaki, Takuji Shirasawa, Takahiko Shimizu

**Affiliations:** ^1^Department of Advanced Aging Medicine, Chiba University Graduate School of Medicine, Inohana, Chuo-ku, Chiba 260-8670, Japan; ^2^Department of Orthopedics, Juntendo University Graduate School of Medicine, Bunkyo-ku, Tokyo 113-0033, Japan; ^3^Molecular Gerontology, Tokyo Metropolitan Institute of Gerontology, Itabashi-ku, Tokyo 173-0015, Japan; ^4^Department of Radiation Biosciences, Faculty of Pharmaceutical Sciences, Tokyo University of Science, 2641 Yamazaki, Noda, Chiba 278-8510, Japan; ^5^Department of Medical Engineering and Technology, Kitasato University School of Allied of Health Science, Kitasato, Sagamihara, Kanagawa 252-0373, Japan; ^6^Redox Biology, Tokyo Metropolitan Institute of Gerontology, Itabashi-ku, Tokyo 173-0015, Japan; ^7^Department of Ageing Control Medicine, Juntendo University Graduate School of Medicine, Bunkyo-ku, Tokyo 113-0033, Japan

## Abstract

Oxidative stress is believed to greatly contribute to the pathogenesis of various diseases, including neurodegeneration. Impairment of mitochondrial energy production and increased mitochondrial oxidative damage are considered early pathological events that lead to neurodegeneration. Manganese superoxide dismutase (Mn-SOD, SOD2) is a mitochondrial antioxidant enzyme that converts toxic superoxide to hydrogen peroxide. To investigate the pathological role of mitochondrial oxidative stress in the central nervous system, we generated brain-specific SOD2-deficient mice (B-*Sod2* ^−/−^) using *nestin-Cre-loxp* system. B-*Sod2* ^−/−^ showed perinatal death, along with severe growth retardation. Interestingly, these mice exhibited spongiform neurodegeneration in motor cortex, hippocampus, and brainstem, accompanied by gliosis. In addition, the mutant mice had markedly decreased mitochondrial complex II activity, but not complex I or IV, in the brain based on enzyme histochemistry. Furthermore, brain lipid peroxidation was significantly increased in the B-*Sod2* ^−/−^, without any compensatory alterations of the activities of other antioxidative enzymes, such as catalase or glutathione peroxidase. These results suggest that SOD2 protects the neural system from oxidative stress in the perinatal stage and is essential for infant survival and central neural function in mice.

## 1. Introduction

Regulation of redox balance is essential throughout life, and dysfunction in the mechanisms underlying the redox balance is believed to be involved in various disease states, including neurodegeneration. Although it remains unclear whether oxidative stress is a major cause or merely a consequence of the cellular dysfunction associated with neurodegenerative diseases [1], accumulating evidence suggests that impaired mitochondrial energy production and increased mitochondrial oxidative damage are early pathological events that lead to neurodegeneration [2]. Superoxide (O_2_^∙−^), one of the reactive oxygen species (ROS) generated by mitochondrial respiration, is involved in a variety of biological processes in central nervous system [3]. The superoxide dismutases (SODs) are enzymes that catalyze the conversion of O_2_^∙−^ to hydrogen peroxide (H_2_O_2_) and help prevent the build-up of toxic O_2_^∙−^. Three SOD isoforms are expressed in mammalian cells: copper/zinc SOD (CuZn-SOD, SOD1), which is located in the cytoplasm [4]; manganese SOD (Mn-SOD, SOD2), which is localized in the mitochondrial matrix [5]; and extracellular SOD (EC-SOD, SOD3) [6]. Together, these enzymes are responsible for protecting cells and tissues from ROS generated from endogenous and exogenous sources. Mitochondria are both a major source of ROS and a major target of ROS-induced cellular injury. Thus, mitochondrial SOD2 is thought to play an important role in cellular defense against oxidative damage by ROS.

In previous studies, two groups independently reported SOD2-deficient mice [7, 8]. The SOD2 deficiency on a CD1 background resulted in neonatal death by day 10 from severe dilated cardiomyopathy, liver dysfunction, and metabolic acidosis [7]. The other SOD2-deficient mice on a mixed genetic background (C57BL/6 and 129/Sv) died by day 18 with fatty liver and neuronal degeneration, particularly in the basal ganglia and brainstem [8]. Therefore, it was impossible to investigate the pathological consequences of oxidative damage in adult tissues using totally SOD2-deficient mice. The authors of these studies also argued that the phenotypes are too complex to sequester the specific aging processes in each tissue of mutant mice* in vivo*. In our previous studies, we have established several types of tissue-specific SOD2-deficient mice to define the phenotypes observed in these systemic SOD2-knockout mice [9–14]. We reported that heart/muscle-specific SOD2-deficient mice (H/M-*Sod2* ^−/−^) show dilated cardiomyopathy involving the excess generation of ROS by mitochondria [12, 15].

In the present study, we successfully generated brain-specific SOD2-deficient mice (B-*Sod2* ^−/−^) using the Cre-loxP system under the control of the nestin promoter and established a murine model for neurodegeneration induced by mitochondrial oxidative stress.

## 2. Results

### 2.1. Generation of B-*Sod2* ^−/−^

**Figure 1:**
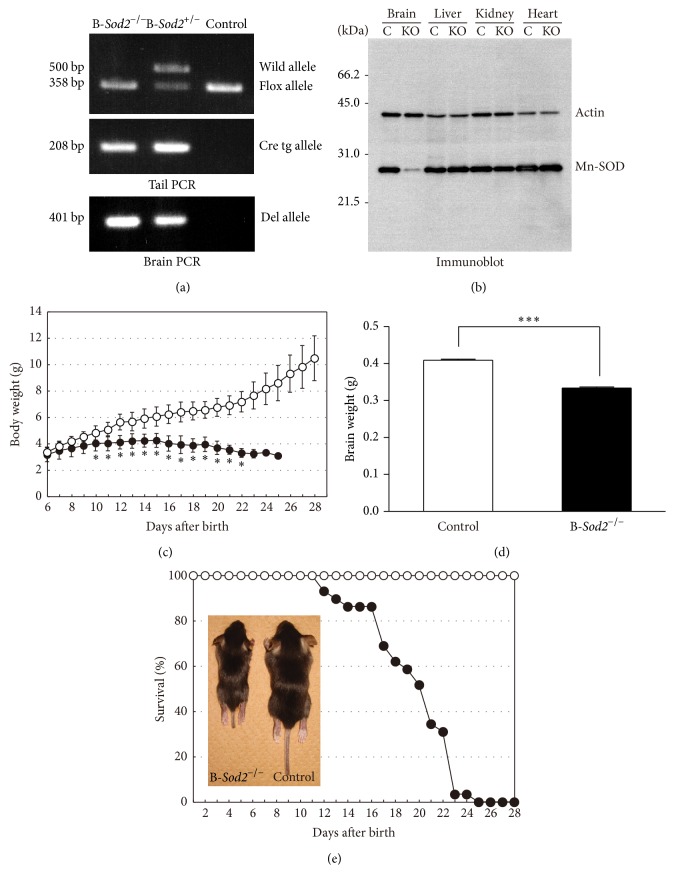
Generation of B-*Sod2* ^−/−^. (a) DNA fragments amplified from the wild (500-bp) or lox allele (358-bp) were detected in tail DNA. The Cre transgene was confirmed by PCR with Cre primers. A 208 bp DNA fragment was detected in knockout and heterozygous mice using Cre PCR primers. A 401 bp DNA fragment amplified from the deletion allele was detected in the brains of knockout and heterozygous mice. (b) The results of Western blot analysis of SOD2 protein in two-week-old B-*Sod2* ^−/−^ and control. Protein extracts from the brain, liver, kidney, and heart of B-*Sod2* ^−/−^ or control were immunoblotted with anti-SOD2 or anti-actin antibodies. (c) The growth rate of body weight increase in B-*Sod2* ^−/−^ (*n* = 24), indicated by closed circle, progressively decreased compared with control (*n* = 29, ^∗^ *P* < 0.05), indicated by open circle. (d) The brain weight of B-*Sod2* ^−/−^ at 16–19 days of age (*n* = 24) was significantly lower than that of brains from age-matched control (*n* = 22, ^∗∗∗^ *P* < 0.005). (e) The cumulative survival of B-*Sod2* ^−/−^ (*n* = 29), indicated by closed circle, and control (*n* = 29), indicated by open circle.

In order to investigate the physiological and pathological role of SOD2 in the brain, we generated B-*Sod2* ^−*/*−^ using the Cre-loxP system. We used nestin-Cre transgenic mice for the selective expression of Cre protein in the neurogenic lineage, such as in neurons, astrocytes, and oligodendrocytes, which are mainly in brain [16], in early prenatal stage in coordination with nestin expression from E7 [17]. As shown in Figure 1, crossbreeding homozygous SOD2-flox mice, which were set as control, with nestin-Cre transgenic mice gave rise to B-*Sod2* ^−*/*−^. Genomic DNA extracted from brain tissues was analyzed by PCR to detect the deleted fragment from the genomic SOD2 gene. Corresponding to the deletion allele, a 401 bp DNA fragment was specifically amplified by PCR from the brains of B-*Sod2* ^−*/*−^ and heterozygous mice, while no fragment was amplified in control mice (Figure 1(a)). Western blot analyses further showed a specific loss of SOD2 expression in the brain, but not in the liver, kidney, or heart of B-*Sod2* ^−*/*−^ compared with control (Figure 1(b)). The slight expression of SOD2 protein in the brains of B-*Sod2* ^−*/*−^ could be derived from nonneurogenic lineage cells, such as microglia or endothelial cells. B-*Sod2* ^−*/*−^ were born in normal Mendelian ratio and in the neonatal stage, and we were unable to find any differences in the visible appearance or body size between B-*Sod2* ^−*/*−^ and control (data not shown). However, at 10 days of age, B-*Sod2* ^−*/*−^ began to exhibit a delayed increase in body weight (Figure 1(c)), and the brain weight of B-*Sod2* ^−*/*−^ was significantly lower than that of control at the age of 16–19 days (Figure 1(d)), probably caused by growth retardation. Finally, the B-*Sod2* ^−*/*−^ began to die from 12 days of age (Figure 1(e)), and all of B-*Sod2* ^−*/*−^ died by 25 days of age, with a median survival rate of 19.6 ± 3.6 days (Figure 1(e)). These results suggest that SOD2 expression in brain is required for the postnatal survival of mice.

### 2.2. B-*Sod2* ^−/−^ Exhibited a Distinct Spongiform Encephalopathy Associated with Gliosis

**Figure 2:**
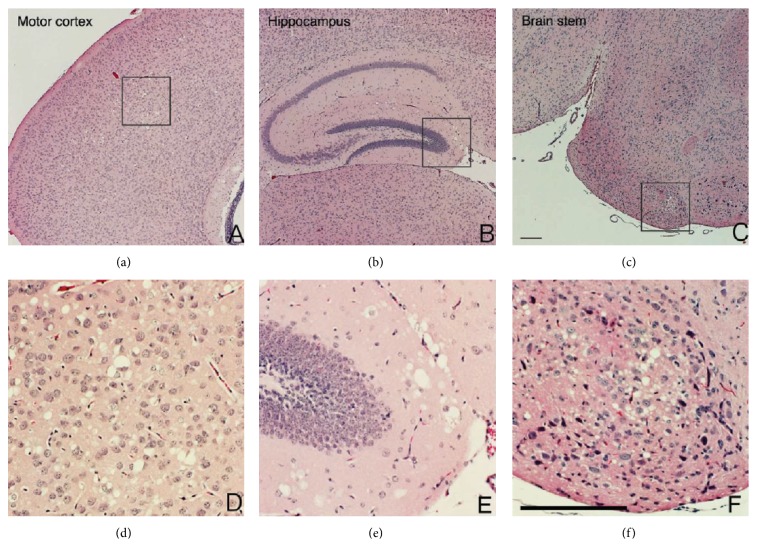
B-*Sod2* ^−/−^ exhibited distinct spongiform encephalopathy in the brain. (a–f) Coronal sections of brains from three-week-old B-*Sod2* ^−/−^. Motor cortex (a, d), hippocampus (b, e), and brain stem (c, f). A large number of vacuoles were morphologically observed in B-*Sod2* ^−/−^. Higher power views (inset) showed neuronal degeneration and malformation with cytoplasmic vacuolization, as well as pleomorphic nuclei (d, e, and f). The scale bars indicate 200 *μ*m.

**Figure 3:**
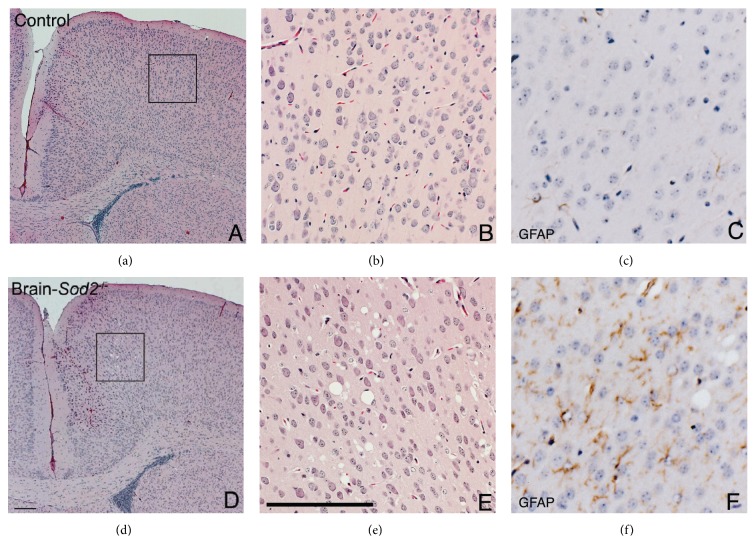
Gliosis was detected in the cortex of B-*Sod2* ^−/−^. Coronal sections of the brains from three-week-old control (a, b) and B-*Sod2* ^−/−^ (d, e) with hematoxylin and eosin staining. Higher power views (inset) showed neuronal vacuolization in B-*Sod2* ^−/−^ (e) but not in control (b). Immunohistochemical staining of sections with anti-GFAP antibody (c and f) revealed enhanced gliosis in B-*Sod2* ^−/−^ (f) but not in control (c). The scale bars indicate 200 *μ*m.

Next, we prepared brain coronal sections from three-week-old B-*Sod2* ^−*/*−^, conducting the histochemical analysis. Hematoxylin and eosin staining revealed vacuolization similar to spongiform encephalopathy selectively in cerebral motor cortex, hippocampus, and brain stem in B-*Sod2* ^−*/*−^, but not in control (Figure 2). This abnormal vacuolization probably resulted from neuronal degeneration due to the susceptibility to oxidative stress. In addition to the vacuolization, we observed that the number of glial fibrillary acidic protein (GFAP) positive cells was increased in cerebral cortex (Figure 3) and hippocampus (data not shown) in B-*Sod2* ^−*/*−^ by immunohistochemical analysis, thus suggesting astrocyte activation. These results suggest that the biological function of SOD2 is essential for preventing neurodegeneration and astrocyte activation.

### 2.3. B-*Sod2* ^−/−^ Showed Specific Defects of Mitochondrial Respiration

**Figure 4:**
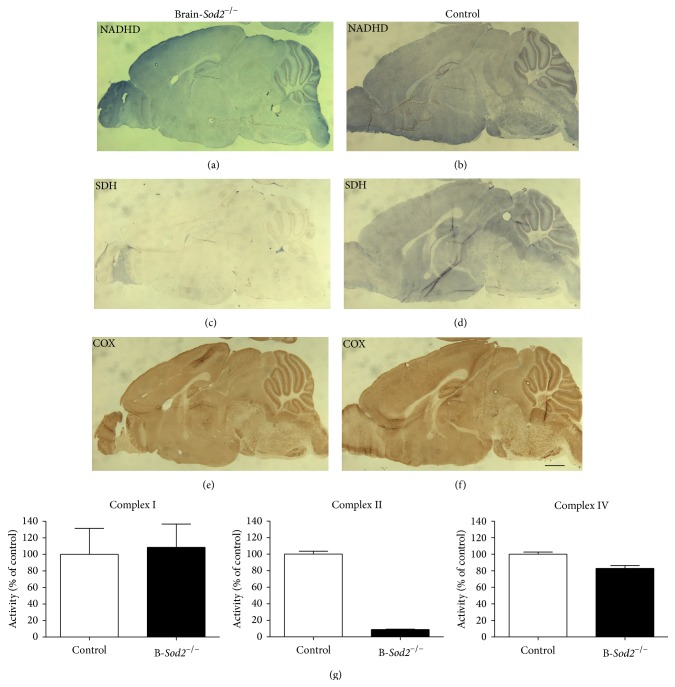
Impaired mitochondrial respiratory activities. Enzymatic histochemical staining for NADHD (top panels) (a, b), SDH (middle panels) (c, d), and COX activities (bottom panels) (e, f) in sagittal sections of brain from three-week-old mice of the indicated genotypes. The scale bars indicate 1 mm. (g) Quantification of enzymatic reactivities of mitochondrial complexes I, II, and IV.

To better understand the biochemical alterations involved in pathogenesis of the brain in the mice, we examined the mitochondrial respiratory functions in the brain of B-*Sod2* ^−*/*−^ and compared them with control. By an enzymatic histochemical analyses of succinate dehydrogenase (SDH), we assessed the biochemical activity of electron transport complex II in mitochondria on sagittal brain sections from three-week-old B-*Sod2* ^−*/*−^ and control. In the brains of B-*Sod2* ^−*/*−^, mitochondrial complex II activity was hardly detected (Figure 4(c)), while strong staining indicating SDH activity was detected in the brains of control (Figure 4(d)). We also assessed the activity of mitochondrial complexes I (NADH dehydrogenase (NADHD)) and IV (cytochrome c oxidase (COX)) in the brain (Figures 4(a), 4(b), 4(e), and 4(f)) of the mice. The enzymatic activity of mitochondrial complexes I and IV in B-*Sod2* ^−*/*−^ showed strong staining that was comparable to that in the control (Figures 4(a), 4(b), 4(e), and 4(f)). These data clearly suggest the selective loss of enzymatic activity of mitochondrial complex II in the brains of B-*Sod2* ^−*/*−^.

### 2.4. Antioxidant Enzyme Activities, Glutathione Levels, and Lipid Peroxidation Levels

**Figure 5:**
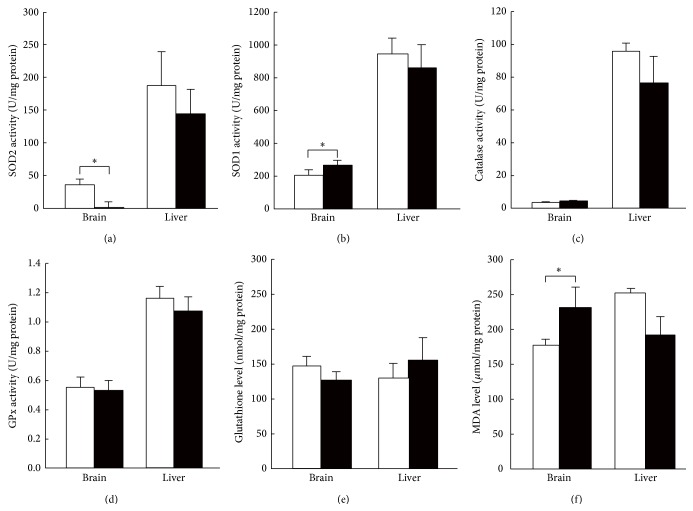
The antioxidative enzyme activities and glutathione and lipid peroxidation levels in B-*Sod2* ^−/−^ indicated by closed column, and control, indicated by open column. (a) SOD2, (b) SOD1, (c) catalase, (d) GPx activities, (e) glutathione levels, and (f) MDA levels were measured in brain and liver lysates of B-*Sod2* ^−/−^ (*n* = 5) and control (*n* = 5) at 3 weeks of age. White and black columns indicate control and B-*Sod2* ^−/−^, respectively (^∗^ *P* < 0.05).

To investigate whether there was a compensatory mechanism for SOD2 deficiency, we measured the activities of antioxidant enzymes in the tissues from B-*Sod2* ^−/−^. Although the SOD2 activity was completely depleted, the compensative increase in SOD1 activity was minimal (Figures 5(a) and 5(b)). We next measured the activities of H_2_O_2_ reductases, such as catalase and glutathione peroxidase (GPx), as well as the levels of glutathione, an endogenous antioxidative substance, but found no significant change in the activities of these enzymes or in the levels of the antioxidants (Figures 5(c)–5(e)). We also measured the amount of malondialdehyde (MDA) as a marker of lipid peroxidation by O_2_^∙−^ in brain lysates. A higher level of MDA was detected in brain extracts from B-*Sod2* ^−/−^ than that from control (Figure 5(f)), indicating that oxidative stress-associated damage accumulated in the brain as a result of SOD2 deficiency.

## 3. Discussion

Deregulation of the redox balance is thought to be involved in aging and in the pathogenesis of various diseases. We recently reported that brain slices from global SOD2-knockout mice exhibited increased amount of ROS after hypoxia-reoxygenation stress, suggesting that SOD2 plays a central role in ROS elimination in* ex vivo* model [18]. In order to elucidate the significance of SOD2 in redox homeostasis* in vivo*, many studies on SOD2-deficient mice have been ongoing in our lab and by other groups. In previous studies, two groups independently developed SOD2-deficient mice, and both lines were reported to show perinatal death, suggesting the physiological and pathological importance of SOD2 [7, 8, 19, 20]. So far, although several lines of tissue or cell-type specific SOD2-knockout mice generated by means of Cre-loxP system have been analyzed [9–14], the cause(s) of the early-stage death was unclear. Since SOD2-deficient mice were reported to manifest lipid accumulation and atypical patterns of glycogen deposition in the liver [7, 8], we generated liver-specific SOD2-knockout mice (liver-*Sod2* ^−/−^) by crossbreeding with SOD2-flox mice and albumin-Cre transgenic mice, in which the Cre transgene is controlled by the enhancer/promoter of the albumin gene so that Cre recombinase is specifically expressed in hepatocytes [9, 10]. Unexpectedly, however, liver-*Sod2* ^−/−^ did not show any obvious morphological changes or early death [9, 10]. Next, we generated and analyzed H/M-*Sod2* ^−/−^, in which SOD2 was selectively depleted in muscle tissues. H/M-*Sod2* ^−/−^ exhibited cardiac enlargement similar to the total SOD2-deficient mice and die by 6 months of age [12]. However, the median survival was about 4 months [12], which was longer than the life span of SOD2 totally depleted mice. Furthermore, we produced skeletal muscle-specific SOD2-knockout utilizing Cre-loxP system under the control of human skeletal actin promoter [13]. These mutant mice exhibited no shortening of life span in spite of severe disturbance in their exercise activity [13]. Although SOD2 total knockout mice showed severe phenotypes in the liver, heart, and skeletal muscle, our findings suggested that there is no direct involvement of SOD2 depletion in these tissues in perinatal death. In this study, we generated B-*Sod2* ^−/−^ by using nestin-Cre transgenic mice, in which Cre protein is selectively expressed in neurogenic lineage [16]. We found that B-*Sod2* ^−/−^ exhibited perinatal death, reproducing the phenotype of the total knockout mice. This suggests that SOD2 in the brain is essential for perinatal survival via protection against oxidative stress.

However, the mechanisms of perinatal death induced by brain-specific SOD2 deletion have not been understood. It is possible that the spongiform encephalopathy observed in B-*Sod2* ^−/−^ brain could cause early death. For example, in cases of prion disease, including bovine spongiform encephalopathy (BSE) in cattle and Creutzfeldt-Jakob disease (CJD) in humans, spongiform neurodegeneration was observed, which was followed by death [21, 22]. In a genetically manipulated animal model, Attractin (Atrn) or Mahogunin mutant mice also developed hippocampal progressive spongiform degeneration, but this was not fatal [23, 24]. The involvement of spongiform neurodegeneration in death is controversial. In the brains of B-*Sod2* ^−/−^, spongiform neuronal loss was observed in restricted area, such as brain stem, cerebral cortex, and hippocampus. Among these regions, brain stem includes the central control for autonomic nervous system. Therefore, spongiform neurodegeneration in brain stem might cause critical dysfunction of autonomic nervous system, leading to perinatal death. However, we cannot exclude the possibility that spongiform encephalopathy is unrelated to the death of the mice. Several studies on heterozygote SOD2-knockout mice, which do not exhibit neuronal loss, showed impaired neuronal transduction [25]. This suggests that the oxidative stress induced by SOD2 deficiency may lead to neuronal dysfunction in some region(s) that are indispensable for survival. The production and analysis of region-specific or stage-specific SOD2 conditional knockout mice could provide a powerful tool for detailed investigations of the mechanism(s) underlying the perinatal death induced by B-*Sod2* ^−/−^.

B-*Sod2* ^−/−^ were born in normal Mendelian ratio and exhibited no abnormalities in visible appearance, suggesting that SOD2 deficiency in brain has no influence on prenatal or neonatal stage. This could be because oxygen supply for infants through placenta is of lower level than that through respiration after birth. Although B-*Sod2* ^−/−^ exhibited severe spongiform degeneration in the brain, no structural abnormalities were observed. This suggests that SOD2 in the brain plays essential roles in the perinatal stage, but not in the early developmental stage. Interestingly, neuronal degeneration was observed in selected regions, such as cerebral motor cortex, hippocampus, and brain stem, although mitochondrial dysfunction resulting from SOD2 deletion was observed in the whole brain. Misawa et al. previously generated postnatal motor neuron-specific SOD2-knockout mice by crossbreeding SOD2-flox mice and VAChT-Cre transgenic mice, in which Cre expression is restricted to the postnatal somatomotor neurons [11]. Compared to B-*Sod2* ^−/−^, motor neuron-specific SOD2-knockout mice showed the loss of SOD2 expression specifically in a subset of somatomotor neurons and, surprisingly, showed no reduction of the number of neurons up to one year after birth, even though extensive histological examinations were performed [11]. These data suggested that postnatal motor neurons are resistant to mitochondrial oxidative stress. On the other hand, we found that dopaminergic neuron-specific SOD2-deficient mice, generated by crossbreeding SOD2-flox mice and tyrosine hydroxylase (TH) Cre mice, exhibited severe loss of dopaminergic neurons accompanied by motor dysfunction (unpublished results). Dopaminergic neurons are highly sensitive to oxidative stress, because these neurons contain reactive quinone, which is converted from intrinsic dopamine through autooxidation [26, 27]. Together, these findings suggest that regional selectivity of spongiform neurodegeneration in B-*Sod2* ^−/−^ reflects the local vulnerability to mitochondrial superoxide.

Since abnormal glioses were observed in the brains of B-*Sod2* ^−/−^, there is a possibility that astrocyte activation induced neurodegeneration. Astrocytes have more potent defense systems against oxidative stress, including NF-E2-related factor 2- (Nrf2-) antioxidant response element (ARE) pathway, than neurons [28]. Astrocytes that are exposed to oxidative stress at physiological levels activate Nrf2-ARE pathway to increase the productions of antioxidants, such as glutathione [28]. Glutathione generated from astrocytes protects not only astrocytes themselves, but also neurons as a secreted protective mediator [28]. On the other hand, under the pathological conditions, such as exposure to excessive levels of oxidative stress or exposure to amyloid *β*, astrocytes are activated and produce neurotoxic cytokines [29]. In the B-*Sod2* ^−/−^, pathological levels of oxidative stress could induce astrocyte activation and the subsequent secretion of large amounts of toxic cytokines, thus damaging neurons. Furthermore, Liang et al. reported that SOD2 heterozygous knockout mice exhibited epilepsy via astrocyte dysfunction [30]. They showed that a reduction of the SOD2 level induced the downregulation of glutamate transporter 1 (GLT-1) and glutamate-aspartate transporter (GLAST), both of which are glutamate transporters that clear glutamate from synapse, which increased the levels of glutamate at the synapse and led to epileptic conditions [30]. This is in agreement with the evidence that patients suffering from genetic mitochondrial disease often have epilepsy. In the brains of B-*Sod2* ^−/−^, the potently downregulated expression of GLT-1 and GLAST could induce a resultant increase in the glutamate concentration at synapse, leading to excitotoxicity.

In this study, the activity of mitochondrial complex II was dramatically decreased by SOD2 deficiency, in spite of the fact that there were no obvious changes in the activities of complex I or IV in the brain. Importantly, the selective downregulation of SDH activity has also been observed in cardiac and somatic muscle tissues in H/M-*Sod2* ^−/−^ [12, 13], suggesting that mitochondrial complex II plays a role as an oxidative sensor localized on electron transport system, as well as citrate cycle, considering that SDH couples electron transport system and citrate cycle. Of note, SOD2 deficiency induced only weak levels of compensatory activity in the antioxidant system. These results suggest that most of the mitochondrial oxidative stress is sequestrated in mitochondria and only a little of it leaks to the cytoplasm and is cleared by SOD1, which is localized in cytosol.

## 4. Experimental Section

### 4.1. Animals

The generation of SOD2-flox mice was described previously [9]. The SOD2-flox mice were backcrossed to C57BL6/CrSlc mice for five or six generations. In order to investigate the physiological role of SOD2 in the central nervous system, we crossbreed SOD2-flox mice with nestin-Cre transgenic mice (Jax #3771, The Jackson Laboratory, Bar Harbor, USA). In nestin-Cre transgenics, the Cre transgene is controlled by the promoter and intronic enhancer elements of the rat nestin gene, so that Cre recombinase is specifically expressed in embryonic neural progenitors [16]. The crossbreeding of SOD2-flox mice with nestin-Cre mice gave rise to B-*Sod2* ^−/−^. Genotyping of the Cre transgene and the SOD2-flox allele was performed by PCR using genomic DNA isolated from the tail tip. The primers used to identify carriers of the nestin-Cre transgene were 5′-TTC CAG CTA GAG AGA CTG AGT CCC-3′ and 5′-TCG ACC AGT TTA GTT ACC C-3′ and those used to identify SOD2-flox allele were 5′-TTA GGG CTC AGG TTT GTC CAG AA-3′, 5′-CGA GGG GCA TCT AGT GGA GAA-3′, and 5′-AGC TTG GCT GGA CGT AA-3′. The deleted alleles were confirmed as described in a previous study [9]. Mice were maintained and studied according to protocols approved by the Animal Care Committee of the Tokyo Metropolitan Institute of Gerontology.

### 4.2. Western Blot Analysis

Western blot analysis was performed with brain, liver, kidney, and heart homogenates (5 *μ*g each) as previously described [9, 10]. Antibodies against SOD2 (1 : 10,000; #SOD-111, Enzo Lifesciences (StressGen), Victoria, Canada) and actin (1 : 100; #A2066, Sigma-Aldrich, St. Louis, MO, USA) were used.

### 4.3. Histological and Histochemical Studies

The organs were dissected, fixed in a 20% formalin neutral buffer solution (Wako, Osaka, Japan) overnight, embedded in paraffin, and sectioned on a microtome following standard techniques. Hematoxylin and eosin staining was performed as described previously [12]. Immunohistochemical staining of brain sections was performed with anti-GFAP antibody (#G9269, Sigma-Aldrich) and VECTASTAIN ABC Elite kit (#PK-6101, Vector Laboratories, Burlingame, CA, USA) according to the manufacturer's protocol.

For enzymatic histochemical staining, tissues were frozen in isopentane in liquid nitrogen and embedded in OTC compound on dry ice. Sections were cut into 8 *μ*m thick sections and were mounted on silane-coated slide glasses. Frozen sections were dried and incubated in 0.1 M Tris-Cl (pH 7.4), 1 mg/mL nitro blue tetrazolium, and 0.1 mg/mL *β*-NADH to assess NADHD (mitochondrial complex I) activity; 50 mM phosphate buffered saline (PBS) (pH 7.4), 84 mM succinate acid, 0.2 mM phenazine methasulfate, 2 mg/mL nitro blue tetrazolium, and 4.5 mM EDTA to assess SDH (mitochondrial complex II) activity; or 50 mM PBS (pH 7.4), 1.0 mg/mL 3,3′-diaminobenzidine, 24 U/mL catalase (Wako), 1 mg/mL cytochrome* c* (Wako), and 75 mg/mL sucrose to assess COX (mitochondrial complex IV) activity. These incubations were performed in the dark at room temperature for 20 min. Quantification was performed by image analyzing software, Leica QWin V3.

### 4.4. Determination of SOD, Catalase, GPx Activities, and Glutathione Content

Tissues from two-week-old mice were homogenized in 0.1 M PBS, pH 7.5, containing 5 mM EDTA, and were centrifuged at 15,000 ×g for 30 min. The supernatant was used to assess the antioxidant enzyme activities. SOD activity was measured by NBT assay [31]. The enzymatic activity was expressed in U/mg of protein. To determine SOD2 activity, lysates were treated with 1 mM KCN to inactivate SOD1. The protein concentration was measured by Lowry method using BSA as a standard. For catalase activity assay, the supernatant was incubated with 9 mM H_2_O_2_, 0.25 mM EDTA, 50 mM Tris-HCl (pH 7.5), and 50 mM PBS at room temperature. Catalase activity was measured by the decrease in H_2_O_2_ absorbance at 240 nm and was expressed as U/mg protein. For GPx activity assay, the supernatant was incubated with 1 U/mL glutathione reductase, 2 mM glutathione, 0.2 mM NADPH, 70 *μ*M t-butyl hydroperoxide, 0.05 mM EDTA, and 10 mM Tris-HCl (pH 7.5) at room temperature. GPx activity was measured by the decrease of NADPH absorbance at 340 nm and was expressed as U/mg protein.

To determine the glutathione content, the supernatant was added with an equal volume of 10% trichloroacetic acid (TCA) to remove acid-insoluble materials. The acid-soluble fraction was extracted with ether to remove residual TCA. The fraction (25 *μ*L) was mixed with 250 *μ*L of 1 mM 5,5′-dithiobis-(2-nitrobenzoic acid), 733 *μ*L of 0.3 mM NADPH, and 10 *μ*L of 2 U/mL glutathione reductase. The rate of change in the absorbance was measured at 412 nm. The glutathione concentration was calculated as nmol/mg protein.

### 4.5. Measurement of Lipid Peroxidation

Lipid peroxidation in tissues homogenates was measured as MDA by the thiobarbituric assay [32]. The MDA level of homogenates was calculated as *μ*mol/mg protein.

### 4.6. Statistical Analysis

We analyzed the data using the unpaired* t*-test and considered *P* values < 0.05 to be statistically significant. The data are expressed as the means plus or minus SD.

## 5. Conclusions

SOD2 null mice have been known to have short survival. However, it was not clear why they died during the perinatal phase. Our findings suggest that SOD2 protects the neural system from postpartum ROS injuries in neonates, and SOD2 has essential roles in perinatal survival and in the central neural functions in mice. Furthermore, we propose that spongiform encephalopathy, along with inflammation, is the major cause of death in B-*Sod2* ^−/−^, and the activity of neuronal SOD2 could not be compensated for by other antioxidative systems. These results suggest that accumulation of intrinsic ROS produced from mitochondria induces severe damage to neuronal system.
